# Female genital schistosomiasis is a neglected public health problem in Tanzania: Evidence from a scoping review

**DOI:** 10.1371/journal.pntd.0011954

**Published:** 2024-03-11

**Authors:** Gladys Mbwanji, Humphrey D. Mazigo, Jane K. Maganga, Jennifer A. Downs

**Affiliations:** 1 Department of Parasitology, School of Medicine, Catholic University of Health and Allied Sciences, Mwanza, Tanzania; 2 School of Public Health, Dean’s Office, Catholic University of Health and Allied Sciences, Mwanza, Tanzania; 3 National Institute for Medical Research, Mwanza, Tanzania; 4 Mwanza Intervention Trials Unit, Mwanza, Tanzania; 5 Bugando Medical Centre, Mwanza, Tanzania; 6 Center for Global Health, Weill Cornell Medicine, New York, New York, United States of America; Federal University of Agriculture Abeokuta, NIGERIA

## Abstract

*Schistosoma haematobium*, the parasite that causes urogenital schistosomiasis, is widely prevalent in Tanzania. In addition to well-known effects on the urinary tract, *S*. *haematobium* also causes clinically- evident damage to the reproductive tract in approximately half of infected women, which is known as female genital schistosomiasis (FGS). FGS has major gynecologic and social consequences on women’s reproductive health, yet little information is available regarding FGS in Tanzania. To cover that gap, we conducted the present scoping review to examine the epidemiology of FGS in Tanzania (both in the mainland and Zanzibar island) and to make recommendations for future work in this area. The available evidence from community-based and hospital-based retrospective studies indicates that FGS is a significant health problem in the country. Very few community-based studies have been reported from mainland Tanzania, and Zanzibar. Our review highlights the scarcity of efforts to address FGS in Tanzania and the need for additional community-based studies. The studies will help us understand the true burden of the disease nationwide, to assess the impact of praziquantel on FGS lesions, and to address social and mental health in relation to FGS. This review emphasizes integration of delivery of FGS related services in primary health care systems through the reproductive health clinics which covers sexually transmitted infections, HIV and cervical cancer screening. These actions are essential if this neglected gynecological disease is to be addressed in Tanzania.

## Author summary

Female genital schistosomiasis (FGS) caused by *Schistosoma haematobium* affects mostly the reproductive tract of girls and women in rural and marginalized communities. This disease has been largely neglected by the schistosomiasis endemic communities and public health professionals. The present review aimed at assessing the evidence/epidemiology of FGS among women/girls in Tanzania.

Furthermore, the review assessed the availability of information, published literature on FGS including comorbidities that address FGS in Tanzania mainland and Zanzibar islands. The evidence generated was important to inform the need to address FGS key gaps among researchers, healthcare workers and communities. Overall, the findings indicated that the knowledge of FGS was lacking among the endemic communities and healthcare workers. Findings from this review have shown the available gaps in literature on FGS in Tanzania, from very few community-and-hospitals based studies reported from mainland Tanzania, and Zanzibar. To address this gap, further research is essential to understanding the true burden of disease-associated morbidity, to assessing the impact of single dose praziquantel in FGS lesions, to understanding mental health in relation to FGS, and to integrating delivery of FGS related services in primary health care systems.

## Introduction

Schistosomiasis is prevalent in tropical and subtropical areas, especially in poor communities with limited access to potable water or adequate sanitation [1]. The infection is common in rural and underdeveloped urban areas where communities rely on open surface water sources such as rivers, streams and lakes for work or daily chores [2]. It is estimated that >250 million people require preventive treatment for schistosomiasis worldwide and over 90% of these live in Africa [3,4]. In sub- Saharan Africa, two species of schistosomes are highly prevalent: *Schistosoma mansoni* that causes intestinal schistosomiasis and *Schistosoma haematobium* that causes urogenital schistosomiasis [1]. Approximately two-thirds of cases on the African continent are caused by *S*. *haematobium* [5]. Urogenital schistosomiasis can occur in both men and women [6]; however, the current review will focus on *S*. *haematobium* infections in women given the greater morbidity associated with female genital schistosomiasis (FGS), including associations with HIV infection and infertility [7]. At present, FGS is one of the most prevalent but also underrecognized gynecological conditions in Africa, affecting roughly 56 million girls and women [8,9].

For over 120 years, *S*. *haematobium* has been known to cause pathology of the urinary and genital tracts in women, with eggs of the parasites reported in vaginal tissues in Egypt in 1899 [10]. Diagnosis of FGS is challenging due to the lack of a simple test specific to the genital tract [11,12]. It is estimated that 33% to 75% of the women living in schistosomiasis endemic areas have FGS [7].

Women actively excreting *S*. *haematobium* eggs in the urine also have the parasite eggs in their uterine cervix, vagina or vulva [13,14]. At the same time, even in the absence of urinary ova excretion in women, 23%–41% of those with *S*. *haematobium* infection are found to have genital schistosomiasis [7]. Further complicating the diagnosis of FGS, women with *S*. *haematobium* infection do not excrete as many eggs for a given worm burden as do men with *S*. *haematobium* [10].

FGS has been implicated as a major cofactor in the poor reproductive health of many women in Africa. It can cause infertility, menstrual and pregnancy complications, genital lesions, pain and bleeding from intercourse, anaemia, genital itching, and genital discharge [15]. On a macroscopic level, FGS has been associated with genital mucosal changes, such as sandy patches and pathological blood vessels [16,17] as well as with genital immunological changes [17,18] that may increase women’s susceptibility to infections such as HIV.

After Nigeria, Tanzania ranks second-highest in schistosome infections in Africa, with a national prevalence ranging between 12–87% [19]. *S*. *haematobium*, which comprises two-thirds of Tanzanian cases, is endemic throughout the country but its transmission is focalized and heterogeneous in nature [5,20–22]. A high prevalence has been repeatedly documented among school aged children using detection of eggs in urine [21]. The parasite has been studied less frequently among women of reproductive age, and studies of FGS in Tanzania are limited. Even less programmatic data is not available to guide local and regional policy. As a result, control program managers and policy makers do not have data to inform them where the FGS burden is greatest, which populations are most affected, and where and how to implement the highest-yield interventions. To address this gap, the present work reviews the available published articles on FGS throughout Tanzania and provides summaries of the key findings and geographical areas where the study was conducted. Studies that address the overlap of FGS with other reproductive health issues are included.

## Methods

### 1. Study design and research question

We conducted a review of literature to address two key research questions: Is there evidence of female genital schistosomiasis in Tanzania, and what is known about FGS from studies conducted in Tanzania?

A scoping review was conducted in order to systematically map the research done in the country, as well as to identify any existing gaps in knowledge. We sought to consider these research questions from a public health perspective in order to guide policy makers in Tanzania.

### 2. Search strategy

To identify potentially relevant papers a full systematic electronic search of literature on PubMed and Google Scholar were used to compile a list of English language papers. It was carried out using predefined search terms for subject headings and text words. In addition, Boolean operators, Medical Subject Heading (MeSH) and non- MeSH terms were also used under the following themes: Epidemiology, AND female genital schistosomiasis, AND comorbidities AND neglected tropical diseases AND Tanzania or Zanzibar. Search terms for subject headings and for text words were developed for four (4) search elements: FGS, comorbidities, epidemiology, and Tanzania. The search terms used for PubMed and Google scholar to identify FGS in Tanzania are provided in S1 Table.

These searches were limited to peer reviewed articles published between 1981 and June 2022 and to studies conducted in human participants only. This systematic approach was done to minimize bias and random errors. Masters and PhD theses available from institutional websites and libraries were not included in this search. All data searches were performed by 4 independent authors (GCM, HDM, JKM, JD). Duplicate citations from the multiple databases were removed.

### 3. Eligibility screening

After the searches were carried out, peer reviewed papers meeting the eligibility criteria were applied by two reviewers (GCM, JKM). Search results were screened using pre-defined criteria, conforming to the Population/Participants, Interventions/exposures, Comparison, Outcome, Setting/Study design (PICOS) framework [23]. Title and abstracts of all search results were screened for eligibility by the reviewers based on prespecified eligibility criteria (S2 Table). Articles that did not meet inclusion criteria were excluded. Studies published in non-English languages were excluded. Quantitative, and qualitative studies were included in order to consider different aspects of measuring female genital schistosomiasis burden.

### 4. Data extraction method

Data were extracted by two authors (GCM, JD) using a standardized extraction form. Extracted data included: publication details, study setting, study design, study population, evaluated outcomes, results and findings, and authors’ conclusions (S3 Table). Tabulated results data were independently extracted by the two reviewers (GCM, HDM), discussed the results and continued charting the results in the data charting form.

### 5. Quality assessment

The in-depth demonstration of the burden of FGS in Tanzania is lacking. To reduce the risk of bias in the eligible studies, a quality assessment was carried out using a risk of bias tool for observational data and classifying each domain into low, high or unclear risk of bias. Five domains were considered: Selection bias, missing data, misclassification of outcome, misclassification of exposures, confounding and statistical analysis approach (S4 Table).

## Results

**Fig 1:**
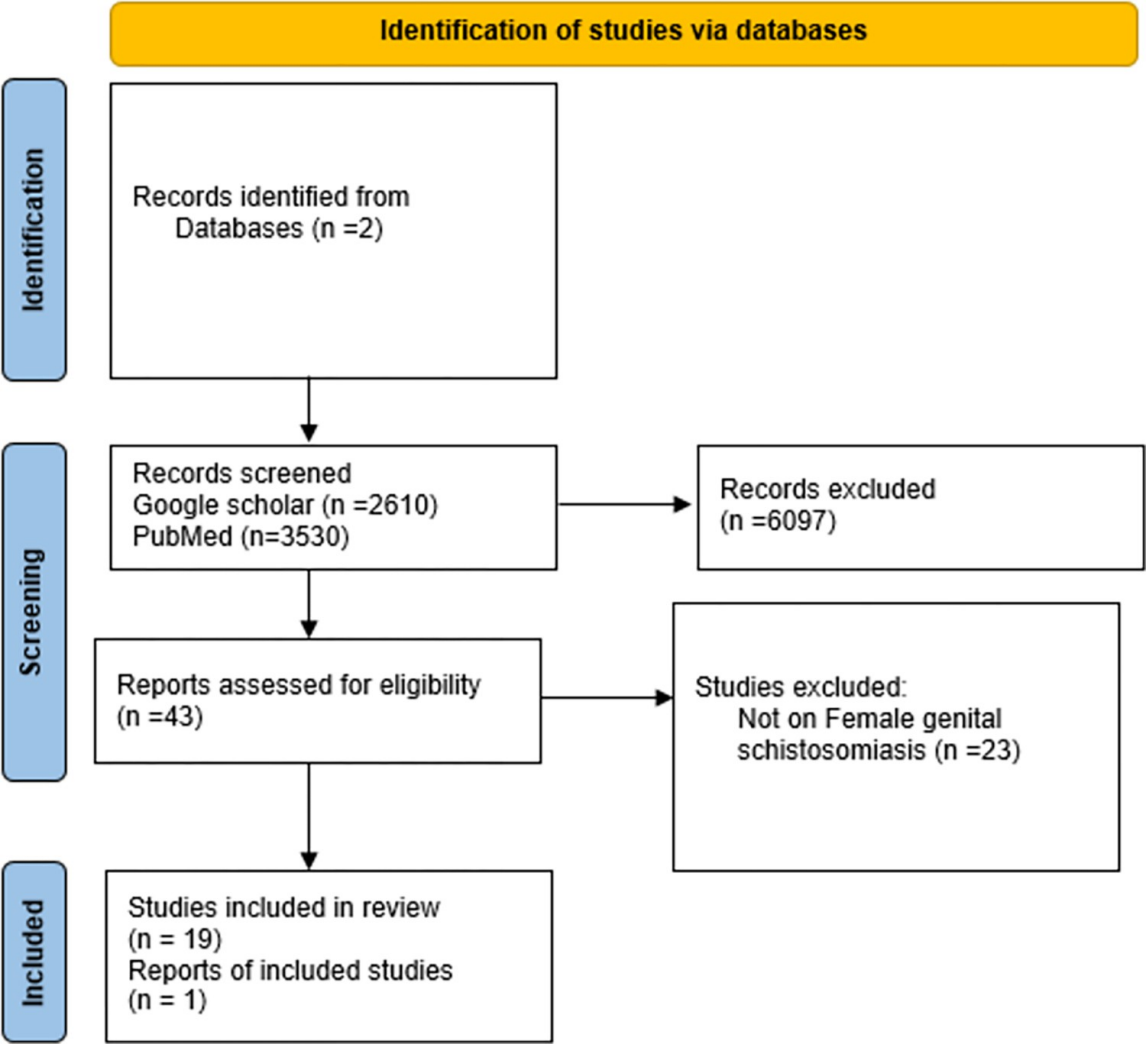
Articles identified, screened, and included in the review.

Of 6,140 records identified from the PubMed and google scholar databases (S1 Table), only 20 papers met the eligibility criteria and were therefore included in this review as shown in Fig 1. Of the 20 papers included in the review we grouped articles as community based epidemiologic studies, hospital based histopathologic studies, case reports, FGS diagnostic studies and qualitative studies as shown in Table 1 below.

**Table 1:** Overview of all studies included in the scoping review.

Article Title	Author/year	Study design	Sample Size	Geographical Location	*Organism studied*	Major findings
Bilharziasis of the female genital tract in Tanzania	Van Raalte J, 1981 [24]	Hospital based study	170 samples from women with genital tract schistosome infection	Muhimbili Medical Centre	*S*. *haematobium*	• Common clinical findings were warts, nodules, swellings and easily bleeding of the cervix • Organs most frequently affected were the cervix (101) cases, vulva (22), vagina (22) and Fallopian tubes (15) • Granulomata formation more common in Fallopian tubes, ovaries than in cervix, vulva and vagina • 17 carcinomas seen overall; 15 in cervix • No clear association between schistosomiasis and cancer
Vulvar *Schistosoma haematobium* lesion treated with praziquantel	Savioli L, 1990 [25]	Case report	A 9-year-old girl with two polypoid nodular growths of the left labium major, hypertrophy of the labium minor on the same side and several firms enlarged non- tender bilateralinguinal lymph nodes	Pemba Island	*S*. *haematobium*	• Lesions had been gradually enlarging for four months • *S*. *haematobium* eggs were seen in scrapings of one growth examined by light microscopy • Urine examination showed haematuria with over 500 eggs per 10 mls of urine • 6 months after praziquantel treatment all lesions had resolved.
Carcinoma of the uterine cervix associated with schistosomiasis and induced by human papillomaviruses	Moubayed P, 1995 [26]	Hospital based study	31 cervical biopsies of squamous cell carcinoma of the uterine cervix including 10 with schistosomiasis co- infection.	Muhimbili Medical Centre	Schistosomes	• 26 out of 31 study cases revealed a specific hybridization of HPVs with varying density and distribution. A slightly labeling of HPV-16 than -18 was demonstrated. • Of the 10 cancers associated with schistosomes, all positive for HPV. • No statistics presented and no definite conclusion can be made about the relationship between schistosomiasis and carcinoma of the cervix with or without HPV.
Schistosomiasis of the lower reproductive tract without egg excretion in urine	Poggensee G, 1998 [14]	Community based study	543 women aged 15–45 years.	Kilimanjaro Region, Tanzania	*S*. *haematobium*	• Prevalence of urinary schistosomiasis was 40% • 32% (85 out of 263) women had genital schistosomiasis • Urinary and genital schistosomiasis coexisted in 62% of women • 23% had *S*. *haematobium* eggs in the cervix without detectable egg excretion in urine • Hematuria was found in 43% of the FGS cases
Screening of Tanzanian women of childbearing age for urinary schistosomiasis: validity of urine reagent strip readings and self-reported symptoms	Poggensee, G, 2000 [15]	Community based study	303 women, 128 living in high and 175 living in low-risk sites for *S*. *haematobium*	Kilimanjaro region, Tanzania	*S*. *haematobium*	• Prevalence of schistosomiasis was 53% in the high-risk and 4% in the low-risk sites. • Pain while urinating and bloody urine were reported significantly more often by women living in the high-risk site • The frequency of haematuria was more among women excreting *S*. *haematobium* eggs than among those who did not (65% versus 32%). • Negative and positive predictive values of all disease markers were poor in the high-risk site, while the negative predictive values were high in the low-risk sites, >96%. • 70% of bloody urine among those infected could be by *S*. *haematobium* infection; however, by the urine reagent strip, only 54% of the hematuria was attributable to *S*. *haematobium* infection. • A third of the self-reported cases with blood in urine in the general population was attributed to *S*. *haematobium* infection
Female genital schistosomiasis of the lower genital tract: prevalence and disease- associated morbidity in northern Tanzania	Poggensee G, 2000 [13]	Community based study	134 women with proven FGS living in S. haematobium endemic area, 225 endemic referents and 75 non-endemic referents	Mwanga District, Kilimanjaro, Tanzania	*S*. *haematobium*	• 36% of eligible women in the endemic area had urinary schistosomiasis • 37% of eligible women in the endemic area who agreed to gynecologic examination had FGS. • Cervical lesions occurred in 75% of the FGS cases, in 4 women from the same endemic region without proven FGS, and in 36% of women from a nonendemic area
Diagnosis of genital cervical schistosomiasis: comparison of cytological, histopathological and parasitological examination	Poggensee G, 2001 [27]	Diagnostic study	Performance of three different ways of detecting schistosome eggs in cervical tissue were compared in 228 women living in a *S*. *haematobium* endemic area	Northern Tanzania	*S*. *haematobium*	• 49% (n = 112) had schistosome eggs detected by the quantitative compressed biopsy technique [QCBT], 18% (n = 40) by the histological examination of a cervical biopsies and 3% (n = 16) by the cytological examination of cervical smears. • The QCBT had the highest sensitivity for schistosome egg detection in the genital cervix.
Presence of *Schistosoma mansoni* eggs in the cervix uteri of women in Mwanga District, Tanzania	Poggensee G, 2001 [28]	Community based study	19 women from a community-based study of 359 women:-10 had both *S*. *haematobium* and *S*. *mansoni* eggs in the cervical biopsy -9 had *S*. *mansoni eggs* in cervical biopsy	Mwanga district, Tanzania	*S*. *mansoni*, *S*. *haematobium*	• Reported symptoms included irregular menstruation (intermenstrual bleeding (26%), infertility (37%), and spontaneous abortion (56%) *S*. *mansoni* in cervix was associated with infertility and abortion compared to the endemic controls and *S*. *haematobium F*GS cases • Colposcopy most often showed inflammation and sandy patches
Human papillomavirus, coinfection with *Schistosoma haematobium*, and cervical neoplasia in rural Tanzania	Petry K, 2003 [29]	Hospital based study	109 Tanzanian patients from a schistosomiasis endemic area age matched with 109 German controls for cytology assessment and HPV DNA detection	Southern Tanzania	HPV and *S*. *haematobium*	• Prevalence of HPV DNA using the Hybrid Capture 2 (HC2) was identical for patients and controls (21.5%) • By PCR, prevalence was 34.5% for Tanzanian case and 26.9% for German controls. Reported history of schistosomiasis was associated with a significantly increased risk for infection with high-risk HPV types, although only 6 women had active *S*. *haematobium* infection
Female genital schistosomiasis as an evidence of a neglected cause for reproductive ill- health: a retrospective histopathological study from Tanzania	Swai B, 2006 [30]	Hospital based study	423 organ specimens with histopathologically confirmed schistosomiasis diagnosis	Kilimanjaro Christian Medical Centre (KCMC), Northern Tanzania	Schistosomes	• Genital schistosomiasis was diagnosed in 41.6% (n = 17) of which 115 were specimens from female patients • Most common symptoms were bleeding disorders (48%) ulcers (17%), tumor (20%), lower abdominal pain (11%) and infertility (7%) • Most cases with genital schistosomiasis were diagnosed as cervical tissue (71 cases) • Cervical cancer was confirmed in 13 patients (25%) who specifically requested for a carcinoma diagnosis • Vulval/labial schistosomiasis was seen in specimens of young women • Infertility was reported in four patients with schistosomiasis
Exploring the feasibility and possible efficacy of mass treatment and education of young females as schistosomiasis influences the HIV epidemic	Lillerud LE, 2010 [31]	Community based study	5-20-year-old-girls	Rural Tanzania	*S*. *haematobium*	• Mass treatment for gynaecological schistosomiasis should be done in collaboration with the school system as a joint venture with the health system in order to reach non-enrolled girls who may be at particular risk. • Research is still needed to assess the preventive effect of treatment on genital lesions and on HIV incidence.
Diagnosis of Female Genital Schistosomiasis by Colposcopy: Feasibility and Options under Conditions of Sub-Saharan Africa	Grothuesmann D, 2010 [32]	Diagnostic study	318, aged 15–45 years	Tanzania	*S*. *haematobium*	• The test validity of colposcopic findings is insufficient. • A standardised process and observational studies to provide further evidence on colposcopy for FGS are necessary.
Urogenital schistosomiasis in women of reproductive age in Tanzania’s Lake Victoria region	Downs JA, 2011 [33]	Community based study	457 women of reproductive age who presented to primary care clinics	North-western Tanzania	*S*. *haematobium*, *S*. *mansoni*	• The overall prevalence of urogenital schistosomiasis was 5% ranging from 0% to 11% • *S*. *haematobium* was higher among women living southern inland villages (10.8%) than those near the lake (3%), (P = 0.002) • Most common symptoms were abdominal pelvic pain (75.5%), menorrhagia (56%), genital itching (54.5%), dysmenorrhea (54.3%), dyspareunia (42.9%) and foul-smelling discharge (31.1%). • In women with FUS, more *S*. *haematobium* eggs were seen in urine (16) than in genital specimens (6) • Prevalence of HIV among women with FUS was 17.4% compared to 5.3% in women without FUS. • No significant difference seen among women with and without FUS in the rates of other sexually transmitted and vaginal infections
Detectable urogenital schistosome DNA and cervical abnormalities 6 months after single-dose praziquantel in women with *Schistosoma haematobium* infection	Downs JA, 2013 [34]	Community based study	Cohort of 33 women with *S*. *haematobium* infection	North-western Tanzania	*S*. *haematobium*	• After 6 months of treatment, no eggs were detected in the women’s urine or cervical samples • 11 (33%) of women had gynaecological abnormalities possibly attributable to schistosomiasis, 6 months praziquantel treatment
Schistosomiasis and Infertility in East Africa	Woodall PA, 2018 [35]	Community based study	study population consisted of women aged 15–49 years, married or in union for at least 5 years, not using a contraceptive, and not presently pregnant.	Tanzania, Kenya, Ethiopia and Uganda	*S*. *haematobium & S*. *mansoni*	Infertility was significantly associated with residence in areas of high *S*. *haematobium* prevalence, compared with both *S*. *haematobium* absence and to equivalent *S*. *mansoni* prevalence. Infertility was not associated with *S*. *mansoni* prevalence.
Altered Cervical Mucosal Gene Expression and Lower Interleukin 15 Levels in Women with *Schistosoma haematobium* Infection but Not in Women with *Schistosoma mansoni*	Dupnik KM, 2019 [36]	Community based study	57 women from *S*. *haematobium*–endemic villages, 18 of whom were infected with *S*. *haematobium;* and 40 women from *S*. *mansoni*–endemic villages, 11 of whom 11 were infected with *S*. *mansoni*.	Northwestern Tanzania	*S*. *haematobium* and *S*. *mansoni*	• 110 genes were differentially expressed in the cervical mucosa of women with and without *S*. *haematobium* infection • IL-15 levels were lower in women with *S*. *haematobium* infection than those without in cervicovaginal lavage (68.2 vs 102.9 pg/mL; *P*adj = .0013) • No differences were found in cervical gene expression in women with and those without *S*. *mansoni* infection. • Additionally, no statistically significant differences in levels of the 27 cytokines were found between the two groups.
Prevalence, Intensity, and Factors Associated with Urogenital Schistosomiasis among Women of Reproductive Age in Mbogwe District Council, Geita Region, Tanzania	Rite E, 2020 [37]	Community based study	426 women of reproductive age (15- 49years) in the selected households who were permanent residents of the Mbogwe District council	North-western Tanzania	*S*. *haematobium*	• 4.5% of participants had urogenital schistosomiasis with a mean egg intensity of 19.5eggs/10mil of urine. • Lower level of education was significantly associated with urogenital schistosomiasis infection.
Detection of *Schistosoma* DNA in genital specimens and urine: A comparison between five female African study populations originating from *S*. *haematobium* and/or *S*. *mansoni* endemic areas.	Pillay P, 2020 [38]	Diagnostic study	933 women from five different study populations (310 women from an *S*. *mansoni* endemic region and 112 women from a nearby *S*. *haematobium* endemic region in Tanzania; 394 women from *S*. *haematobium* endemic regions in South Africa; and 117 women from highly- endemic communities and 38 women who were urine microscopy negative from a low- endemic community Madagascar)	Mwanza, Tanzania, South Africa and Madagascar	*S*. *haematobium* and *S*. *mansoni*	Schistosoma DNA was detected in 13% (120/933) of the CVLs, ranging from 3% in the *S*. *mansoni* Tanzanian endemic region to 61% in the pre-selected Malagasy urine microscopy positive cases. • Detectable Schistosoma DNA in CVL was associated with Schistosoma DNA in urine but not with microscopic detection of eggs in urine or by cytological examination.
Cervicovaginal bacterial communities in reproductive-aged Tanzanian women with *Schistosoma mansoni*, *Schistosoma haematobium*, or without schistosome infection	Bullington B, 2021 [39]	Community based study	39 women with *S*. *mansoni* were compared with 52 uninfected controls, and 16 with *S*. *haematobium* with 27 controls to determine effects of schistosome infection in cervicovaginal microbiota	North-western Tanzania	*S*. *haematobium*, *S*. *mansoni* and cervicovaginal bacterial populations	Presence of *Peptostreptococcus* and *Prevotella timones* more in *S*. *mansoni* infected women compared to control (p = 0.026 and p = 0.048) respectively. High-intensity of *S*. *haematobium* infection and *S*. *mansoni* was associated with various types of cervicovaginal based communities than uninfected controls. There was increased alpha diversity in both *S*. *haematobium* and *S*. *mansoni* infection groups compared to controls at follow up.
“We know about schistosomiasis but we know nothing about FGS”: A qualitative assessment of knowledge gaps about female genital schistosomiasis among communities living in Schistosoma haematobium endemic districts of Zanzibar and Northwestern Tanzania”.	Mazigo H, 2021 [40]	Qualitative study	40 FGDs 37 KIIs	Zanzibar and Northwestern Tanzania	*S*.*haematobium*	• Community members living in two very different areas of Tanzania exhibited major, similar gaps in knowledge about FGS. • Data illustrated a critical need for the national control program to integrate public health education about FGS during the implementation of school- and community-based mass drug administration (MDA) programs and the improvement of water, sanitation and hygiene (WASH) facilities.

### Community based studies

Community-based studies conducted in northern Tanzania [13,14,31,35,36,39] have revealed that FGS is a public health problem among women living in these areas. In a community-based study in northern Tanzania in 1998, 40% of 543 women of child-bearing age were diagnosed with urinary schistosomiasis, and among 263 women who agreed to cervical biopsy, 85 (32%) of them had *S*. *haematobium* eggs seen in wet crushed biopsies [14]. Co-existence of urinary schistosomiasis and FGS was observed in 62% of the women, while 23% of them had eggs in their cervical tissue without detectable eggs in urine samples [14]. In the same setting, in 2000, the overall prevalence of urinary schistosomiasis was 36% among 657 women screened at community level and that of FGS was 37% (134/359) [13]. Cervical lesions occurred in 75% of the FGS cases, with the majority presenting with swollen and disrupted epithelium [13]. One additional report in northern Tanzania revealed that not only *S*. *haematobium* but also *S*. *mansoni* contributed to FGS [28,35] In that report, ~5% of the cervical tissues examined had *S*. *mansoni* eggs and these women complained of irregular menses (41%), intermenstrual bleeding (26%), infertility (37%) and spontaneous abortion (56%) [28]. Of note, this entire body of work in northern Tanzania was conducted over 20 years ago and no further data has been reported from this part of the country.

In north-western Tanzania, the prevalence of *S*. *haematobium* infection was 5% among women screened at the community level, with marked variation from one village to another (from 0%-11% based on urine and cervical microscopy [33]. In this setting, *S*. *haematobium* infection was noted to be associated with HIV infection, with a four- fold increased odds of HIV infection among women diagnosed with FGS. Similarly, FGS was associated with younger age ≤ 35 years [33]. In a community-based intervention study, single dose praziquantel treatment resulted in declining schistosome eggs in urine and schistosome Polymerase Chain Reaction (PCR) values in urine and cervical lavages six months post-treatment, but cervical abnormalities did not improve significantly [34]. A recent population-based study in another district in northwest Tanzania reported that overall prevalence of urogenital schistosomiasis was 4.5% based on detectable eggs in urine samples, with a mean egg intensity of 19.5eggs/10mL [37]. Notably, this study did not include gynaecologic examinations.

Studies in northwest Tanzania have further characterized the co-occurrence of FGS and other diseases of the female reproductive tract, which is complicated given that gynecological symptoms such as abdominal-pelvic pain, menorrhagia, genital itching, dysmenorrhea, dyspareunia and foul-smelling discharge were commonly noted and can be symptoms of FGS or of sexually-transmitted infections [33]. Other community-based studies in the region have investigated immunologic and microbial associations with FGS and documented altered cytokine profiles in cervical lavages of women with *S*. *haematobium* infection and altered cervicovaginal bacterial populations in women with *S*. *mansoni* infection and women with persistent *S*. *haematobium* infection following treatment [28,34]. Other studies have demonstrated that mass treatment for gynaecological schistosomiasis should be done in collaboration with the school system as a joint venture [31]. Does treatment with praziquantel in infected women lead to restoration of cytokine levels and bacterial populations? The answer is still unclear.

### Hospital based studies

Histopathological studies conducted in national and referral hospitals in Tanzania have revealed a significant burden of FGS in women who underwent biopsies or surgery [24,29,30]. At the Muhimbili National hospital in Dar es Salaam, a retrospective study from 1975–1980 analysed a total of 170 specimens involving schistosomiasis of the female genital tract [24]. The commonest clinical manifestations observed were warts, nodules, papillomata, swelling and cervical bleeding [24]. FGS most frequently affected the cervix, followed by the vulva, vagina, and Fallopian tubes [24]. Another retrospective review of histopathological tissues revealed the co-occurrence of the human papillomaviruses (HPVs) and urogenital schistosomiasis in 31 cervical cancer patients and concluded that there was no evidence that the parasite is associated with cervical cancer because all women with *S*. *haematobium* infection also had HPV type 16 or 18 [26]. In southern Tanzania, women who reported a history of urogenital schistosomiasis more frequently had infection with a high-risk HPV type, although the numbers included in this study were small and only 6 women had confirmed *S*. *haematobium* infection [29]. At a consultant hospital in northern Tanzania from 1994–2005, a total of 423 organ specimens were found to contain schistosome ova, 125 (41%) of which were from female genital organs [30]. Predominant symptoms associated with FGS included bleeding disorders, ulcers, tumors, lower abdominal pain, and infertility [30]. Out of 53 cases of suspected cervical cancer, 40 (75%) had severe cervical schistosomiasis and only 13 (25%) had confirmed malignancy [30].

### Case report

In the past four decades, only a single case of FGS involving a 9-year-old girl from Pemba island was published [25]. This girl presented with two polypoid nodular growths of the left labium major and hypertrophy of the left labium minor. *Schistosoma haematobium* eggs were detected both in her urine sample and in a scraping of the genital tissue. Six months after a single dose of praziquantel, she had experienced full resolution of her symptoms.

### Diagnostic studies

Several diagnostic studies focused on improving the detection of genital schistosomiasis at the community level or within hospital settings [27,32,38]. A study from 2001 compared three methods: cytological inspection of a cervical smear, histopathological examination of a preserved biopsy, and parasitological examination of a wet crushed cervical biopsy [27]. Among 228 women, 3% had positive cervical smears, 18% had eggs detected histopathologically, and 49% had schistosome eggs detected in the cervix using the quantitative compressed biopsy technique. It is worthwhile to note that performing a cervical biopsy, except when necessary to rule out cervical cancer, is no longer recommended in areas with endemic HIV infection due to concerns of transiently increasing the risk of HIV acquisition while the cervical mucosa heals.

Urine reagent strip to detect haematuria is a simple indirect tool that has been studied for rapid screening of *S*. *haematobium* infection in endemic areas [27], although false positives can be obtained in cases of urinary tract infections or during menses. In women, reagent urine strips identified haematuria in 65% of those with *S*. *haematobium* eggs in urine [27].

In a three-country study that included 933 women, of whom nearly half were from the Mwanza region of Tanzania, cervicovaginal lavage PCR for *Schistosoma* DNA was significantly associated with detection of *Schistosoma* DNA in urine, demonstrating the utility of real-time PCR to diagnose FGS in endemic areas. Of note, cervicovaginal PCR positivity was not associated with urine egg microscopy, nor with cytologic examination for eggs in genital specimens [27].

### Qualitative studies

A qualitative study was conducted in 2021 on FGS. It focused on gaps in healthcare workers’ knowledge about FGS in Tanzania. Findings from the community-based study demonstrated that most communities living in known *S*. *haematobium* endemic areas of Tanzania had a relatively good knowledge of urogenital schistosomiasis but lacked knowledge of FGS [40].

Misconceptions on the aetiology and modes of transmission of urogenital schistosomiasis and FGS were common. Community members recognized the need for being educated about these diseases. The data emphasized the urgent need for public health interventions to focus on improving community awareness of FGS, which in turn will reduce stigma and improve women and girls’ health seeking behavior when they have genital related lesions.

The findings among health care workers demonstrated the high need for offering education about FGS to fill knowledge gaps. The data suggested that in-service training should cover such topics as identification of women and adolescent girls at risk; symptoms, etiology, modes of transmission, and ways of preventing FGS; management of FGS (including the management of other STIs as part of the differential diagnosis); and mitigating stigma faced by women and girls suffering from FGS both at the healthcare facility settings and in the communities.

## Discussion

The current review indicates that, for the past four decades, there have been sporadic hospital-based retrospective studies, community-based studies, case reports, immunological, and diagnostic studies addressing FGS in Tanzania. However, there is a marked paucity of community-based studies focusing on FGS in many areas of Tanzania, in both Tanzania’s mainland and Tanzania’s islands Pemba and Unguja (Zanzibar). Overall, the current scoping review reveals that FGS occurs in Tanzanian women living in areas of the country with different transmission intensities of *S*. *haematobium* [13,14,33]. Retrospective hospital- based studies at national and zonal consultant hospitals confirm that *S*. *haematobium* eggs have been commonly identified in women’s reproductive tract tissues submitted for pathological examination at histopathology departments for decades. Furthermore, community-based studies conducted in the northern and the north-western part of the country have concluded that FGS is common, though heterogeneously dispersed, in these areas [24,29,30]. We found a severe lack of information on FGS from Unguja and Pemba. Only one case report of FGS involving a young girl was retrieved [25]. This is particularly striking because the two islands are known to be highly endemic for *S*. *haematobium*, with high prevalence of infection in both sexes and a range of age groups [41]. Indeed, multiple studies have worked towards elimination of *S*. *haematobium* from the islands [41,42], but there has been no focus on FGS.

Overall, considering the high prevalence of *S*. *haematobium* infection in Tanzania [21], FGS has been neglected in the country. These findings suggest that routine, repeated MDA or other interventions may be needed to improve the cervical tissue abnormalities caused by FGS in women living in highly endemic areas. However, to date, there is paucity of information on the effects of praziquantel on the gynaecological lesions resulting from *S*. *haematobium* infections. Public health efforts are hindered by lack of reliable up-to-date epidemiological information on prevalence, incidence and geographical distribution of FGS in the country. Community-based studies included in the current review were conducted in only two geographical areas in the country, with that from northern part reported from only a single district of Mwanga and conducted over 20 years ag [13,14]. Given efforts for mass administration of praziquantel in Tanzania and the effects of climate change which highly impacts snail survival [42], up-to-date epidemiological information is urgently needed. We anticipate that current data from across the country will elevate FGS to the Tanzanian national agenda. This will allow FGS not only to be considered for interventions by policy makers, but also to be integrated in improving women’s overall health—reproductive, maternal, and mental health—in Tanzania. One approach to increase the availability of data is to integrate FGS screening, diagnosis, and treatment into women’s reproductive health services in endemic health facilities, as recently recommended in the *Bulletin of the World Health Organization* [43]. This approach will help to generate current epidemiological data from clinical facilities as well as to view women’s health holistically, as experienced by the women themselves. Importantly, this approach will require trained and knowledgeable health workers and availability of appropriate diagnostic equipment. Our team is working to improve knowledge and provide training to local health workers as part of all of our currently ongoing studies of FGS in Tanzania, and further capacity building should be prioritized.

### Limitation

It should be noted that this review was not conducted without limitation. This review only included papers available in google scholar and PubMed. To add to this some articles contained proprietary information which are not publicly available. Furthermore, this scoping review only included studies to June 2022.

### Conclusion

Female Genital Schistosomiasis is a significant problem in Tanzanian women living in *S*. *haematobium transmission* zones; however, up-to-date epidemiological data to guide interventions is severely lacking. The island part of the country has limited data related to FGS available in the public domain. Current efforts to administer single-dose mass praziquantel treatment for schistosomiasis are intensified among school children in Tanzania but not among adolescent girls and women of reproductive age, many of whom are no longer in school. Further complicating this challenge, women with FGS may require multiple doses of praziquantel or other intervention to achieve complete resolution of FGS. Epidemiological and treatment studies are urgently needed. Moreover, further research is essential to understanding the true burden of disease-associated morbidity, to assessing the impact of single dose praziquantel in FGS lesions, to understanding mental health in relation to FGS, and to integrating delivery of FGS related services in primary health care systems. We urge that this most neglected aspect of schistosomiasis, which is itself already a neglected tropical disease, receives the attention that women suffering with FGS deserve.

## Supporting information

S1 Table Search terms and limits used by database. (DOCX)

S2 Table Study eligibility criteria, conforming to PICOS format as per PRISMA recommendations. (DOCX)

S3 Table Data extracted from included publications. (DOCX)

S4 Table Narrative synthesis of quality assessment. (DOC)
