# Supplementary material for: Female genital schistosomiasis is a neglected public health problem in Tanzania: Evidence from a scoping review
Source: PLoS Negl Trop Dis. 2024 Mar 11;18(3):e0011954. doi: 10.1371/journal.pntd.0011954 (PMC10927128; doi:10.1371/journal.pntd.0011954)
Supplement: S1 Table — (DOCX) [file pntd.0011954.s001.docx]

**S1 Table. Search terms and limits used by database**

| **Source** | **Search terms^a,b,^** | **Limits applied** |
| --- | --- | --- |
| PubMED | ""((((((""""female genital schistosomiasis""""[Text Word]) OR (""""genital schistosomiasis""""[Text Word])) OR (FGS[Text Word])) OR (FUS[Text Word])) OR (""""female urogenital schistosomiasis""""[Text Word])) AND ((((((((knowledge*[Text Word]) OR (research*[Text Word])) OR (prevalence*[Text Word])) OR (epidemiology[Text Word])) OR (diagnosis[Text Word])) OR (comorbidit*[Text Word]))) OR (frequenc*[Text Word]))) AND ((((tanzania[Text Word]) OR (zanzibar[Text Word])) OR (north* tanzania[Text Word])) OR (rural Tanzania[Text Word]))""""(""""female genital schistosomiasis""""[Text Word] OR """"genital schistosomiasis""""[Text Word] OR """"FGS""""[Text Word] OR """"FUS""""[Text Word] OR """"female urogenital schistosomiasis""""[Text Word]) AND (""""knowledge*""""[Text Word] OR """"research*""""[Text Word] OR """"prevalence*""""[Text Word] OR """"epidemiology""""[Text Word] OR """"diagnosis""""[Text Word] OR """"comorbidit*""""[Text Word] OR """"frequenc*""""[Text Word]) AND (""""Tanzania""""[Text Word] OR """"zanzibar""""[Text Word] OR (""""north*""""[All Fields] AND """"Tanzania""""[Text Word]) OR """"rural tanzania""""[Text Word])""" | Humans only;  From 1981 – 16 July 2022 |
| Google Scholar | female genital schistosomiasis (Tanzania OR Zanzibar OR Pemba) | Humans only |

FGS: Female Genital Schistosomiasis, FUS: Female urogenital schistosomiasis

^a^General search themes were first established based on the research questions. The themes were then used to map out relevant and appropriate MeSH and non-MeSH search terms that could be used in the database searches.

^b^The operator *** refers to unlimited right-hand truncation. For example, the term ‘comorbidit*’ allows retrieval of citations containing the word ‘comorbidity or ‘comorbidities.
