# Supplementary material for: Female genital schistosomiasis is a neglected public health problem in Tanzania: Evidence from a scoping review
Source: PLoS Negl Trop Dis. 2024 Mar 11;18(3):e0011954. doi: 10.1371/journal.pntd.0011954 (PMC10927128; doi:10.1371/journal.pntd.0011954)
Supplement: S2 Table — (DOCX) [file pntd.0011954.s002.docx]

**S2 Table. Study eligibility criteria, conforming to PICOS format as per PRISMA recommendations^23^**

| **Population or participants** | Sexually active adolescent girls and women from Tanzania aged 15- 49 from either community or hospital settings |
| --- | --- |
| **Intervention or exposure** | All women living near contaminated fresh water source for over 2 years in rural FGS endemic areas of Tanzania, with low income or poor, with/without education, co-mobidities, PZQ efficacy |
| **Outcomes** |  |
| **Setting** | Any setting within Tanzania mainland or Zanzibar |
| **Study designs** | Quantitative, qualitative and mixed-method studies were included in order to consider different aspects of measuring treatment burden. Analytica observational (cross sectional, or cohort studies) that either reported on prevalence, risk factors, diagnosis, epidemiology, or FGS co infection with other diseases provided raw da ta for calculation of effect estimates. Papers were excluded if they did not fit into the conceptual framework of the study, focused on a schistosomiasis, urogenital schistosomiasis. |
| **Language** | Studies in English language were selected. |
| **Exclusion criteria** | Studies not meeting inclusion criteria were excluded. Additionally:   1. Studies that were not in humans. 2. Studies not published between 1981- 2022 (June). |
| **Publication status** | The review included published data. The authors could have considered including a wider range of grey literature (e.g. PhD theses) and unpublished data, and studies published in non-English languages, if they had the resources to do this. |

FGS: female genital schistosomiasis PZQ:praziquantel
