# Supplementary material for: Female genital schistosomiasis is a neglected public health problem in Tanzania: Evidence from a scoping review
Source: PLoS Negl Trop Dis. 2024 Mar 11;18(3):e0011954. doi: 10.1371/journal.pntd.0011954 (PMC10927128; doi:10.1371/journal.pntd.0011954)
Supplement: S3 Table — (DOCX) [file pntd.0011954.s003.docx]

**S3 Table. Data extracted from included publications**

| **Publication details** | Authors  Year of publication |
| --- | --- |
| **Study design** | Study design  Target sample size |
| **Study population** | Number of participants recruited  Age of study participants  Geographical settings |
| **Evaluated outcomes** | Morbidity and comorbidities |
| **Major findings** | Female genital schistosomiasis |
| **Conclusions** | Key conclusions of study authors |
