# Supplementary material for: Female genital schistosomiasis is a neglected public health problem in Tanzania: Evidence from a scoping review
Source: PLoS Negl Trop Dis. 2024 Mar 11;18(3):e0011954. doi: 10.1371/journal.pntd.0011954 (PMC10927128; doi:10.1371/journal.pntd.0011954)
Supplement: S4 Table — (DOC) [file pntd.0011954.s004.doc]

**S4 Table**: **Narrative synthesis of quality assessment**

|  | **Selection bias** | **Outcome mis classfication** | **Exposure mis classificiation** | **Missing data** | **Confounding** | **Statistical analysis approach** |
| --- | --- | --- | --- | --- | --- | --- |
| **Cross sectional studies** |  |  |  |  |  |  |
| Poggensee G, 1998 [1] | U | U | U | U | U | U |
| Poggensee G, 2000 [2] | H | L | L | L | L | L |
| Poggensee G, 2000 [3] | U/L | L | L | L | U | L |
| Poggensee G, 2001 [4] | U/L | L | L | L | U | L |
| Poggensee G, 2001 [5] | L | L | L | L | L | L |
| Dupnik KM, 2019 [6] | L | L | L | L | L | L |
| Rite E, 2020 [7] | L | L | L | U | L | L |
| Bullington B, 2021 [8] | L | L | L | U/L | U/L | L |
| Van Raalte J, 1981 [9] | H | L | L | L | L | L |
| Moubayed P, 1995 [10] | L | L | L | L | L | L |
| Petry K, 2003 [11] | H | H | H | L | L/H | L |
| Pillay P, 2020 [12] | L | L | L | L | L | L |
| Woodall PA, 2018 [13] | L | L | L | L | L | L |
| Lillerud LE, 2010 [14] | L | L | L | L | L | L |
| Grothuesmann D, 2010 [15] | H | U | U | U/L | L | L |
|  |  |  |  |  |  |  |
| **Cohort studies** |  |  |  |  |  |  |
| Swai B, 2006 [16] | L | L | L | L | L | L |
| Downs JA, 2011 [17] | H | L | L | L | L | L |
| Downs JA, 2013 [18] | H | L | L | L | L | L |
|  |  |  |  |  |  |  |
| **Case report** |  |  |  |  |  |  |
| Savioli L,1990 [19] | L | L | L | L | L | L |
|  |  |  |  |  |  |  |
| **Qualitative study** |  |  |  |  |  |  |
| Mazigo H, 2021 [20] | L | L | L | L | L | L |

**S4 Table legends**

1. U- Unknown risk,
2. L-Low risk,
3. H-high risk

**REFERENCES**

1. Poggensee G, Kiwelu I, Saria M, Richter J, Krantz I, Feldmeier H. Schistosomiasis of the lower reproductive tract without egg excretion in urine. Am J Trop Med Hyg [Internet]. 1998 Nov 1 [cited 2023 May 19];59(5):782–3. Available from: https://www.ajtmh.org/view/journals/tpmd/59/5/article-p782.xml

2. Poggensee G, Krantz I, Kiwelu I, Feldmeier H. Screening of Tanzanian women of childbearing age for urinary schistosomiasis: validity of urine reagent strip readings and self-reported symptoms. Bull World Health Organ. 2000;78(4):542–8.

3. Poggensee G, Kiwelu I, Weger V, Göppner D, Diedrich T, Krantz I, et al. Female genital schistosomiasis of the lower genital tract: Prevalence and disease-associated morbidity in Northern Tanzania. J Infect Dis. 2000;181(3):1210–3.

4. Poggensee G, Krantz I, Kiwelu I, Diedrich T, Feldmeier H. Presence of Schistosoma mansoni eggs in the cervix uteri of women in Mwanga District, Tanzania. Trans R Soc Trop Med Hyg. 2001;95(3):299–300.

5. Poggensee G, Sahebali S, Van Marck E, Swai B, Krantz I, Feldmeier H. Diagnosis of genital cervical schistosomiasis: comparison of cytological, histopathological and parasitological examination. Am J Trop Med Hyg. 2001 Sep;65(3):233–6.

6. Dupnik KM, Lee MH, Mishra P, Reust MJ, Colombe S, Haider SR, et al. Altered Cervical Mucosal Gene Expression and Lower Interleukin 15 Levels in Women With Schistosoma haematobium Infection but Not in Women With Schistosoma mansoni Infection. 2019;10065:1777–85.

7. Rite EE, Kapalata SN, Munisi DZ. Prevalence, Intensity, and Factors Associated with Urogenital Schistosomiasis among Women of Reproductive Age in Mbogwe District Council, Geita Region, Tanzania. López Sández CM, editor. Biomed Res Int [Internet]. 2020;5923025. Available from: https://doi.org/10.1155/2020/5923025

8. Bullington BW, Lee MH, Mlingi J, Paul N, Aristide C, Fontana E, et al. Cervicovaginal bacterial communities in reproductive-aged Tanzanian women with Schistosoma mansoni, Schistosoma haematobium, or without schistosome infection. ISME J. 2021 May;15(5):1539–50.

9. Van Raalte JA, Venkataramaiah NR, Shaba JK. Bilharziasis of the female genital tract in Tanzania. East Afr Med J [Internet]. 1981;58(7):543–7. Available from: http://europepmc.org/abstract/MED/7308113

10. Moubayed P, Ziehe A, Peters J, Mwakyoma H, Schmidt D. Carcinoma of the uterine cervix associated with schistosomiasis and induced by human papillomaviruses. Int J Gynaecol Obstet Off organ Int Fed Gynaecol Obstet. 1995 May;49(2):175–9.

11. Petry KU, Scholz U, Hollwitz B, Von Wasielewski R, Meijer CJLM. Human papillomavirus, coinfection with Schistosoma hematobium, and cervical neoplasia in rural Tanzania. Int J Gynecol cancer Off J Int Gynecol Cancer Soc. 2003;13(4):505–9.

12. Pillay P, Downs JA, Changalucha JM, Brienen EAT, Ramarokoto CE, Leutscher PDC, et al. Detection of Schistosoma DNA in genital specimens and urine: A comparison between five female African study populations originating from S. haematobium and/or S. mansoni endemic areas. Acta Trop [Internet]. 2020;204(January):105363. Available from: https://doi.org/10.1016/j.actatropica.2020.105363

13. Woodall PA, Kramer MR. Schistosomiasis and infertility in East Africa. Am J Trop Med Hyg. 2018;98(4):1137–44.

14. Lillerud LE, Stuestoel VM, Hoel RE, Rukeba Z, Kjetland EF. Exploring the feasibility and possible efficacy of mass treatment and education of young females as schistosomiasis influences the HIV epidemic. Arch Gynecol Obstet. 2010 Mar;281(3):455–60.

15. Grothuesmann D. Diagnosis of Female Genital Schistosomiasis by Colposcopy: Feasibility and Options under Conditions of Sub-Saharan Africa. 2010;(March).

16. Swai B, Poggensee G, Mtweve S, Krantz I. Female genital schistosomiasis as an evidence of a neglected cause for reproductive ill-health: A retrospective histopathological study from Tanzania. BMC Infect Dis. 2006;6:1–8.

17. Downs JA, Mguta C, Kaatano GM, Mitchell KB, Bang H, Simplice H, et al. Urogenital schistosomiasis in women of reproductive age in Tanzania’s Lake Victoria region. Am J Trop Med Hyg. 2011;84(3):364–9.

18. Downs JA., Kabangila R, Verweij JJ, Jaka H E. Detectable Urogenital Schistosome DNA and Cervical Abnormalities Six Months after Single-Dose Praziquantel in Women with Schistosoma haematobium Infection. Natl Inst Heal [Internet]. 2013;18(9):1090–6. Available from: https://www.ncbi.nlm.nih.gov/pmc/articles/PMC3624763/pdf/nihms412728.pdf

19. Savioli L, Gabrielli A, Neve H. Vulvar Schistosoma haematobium lesion treated with praziquantel. Trop Doct. 1990 Jan;20(1):45–6.

20. Mazigo HD, Samson A, Lambert VJ, Kosia AL, Ngoma DD, Murphy R, et al. “We know about schistosomiasis but we know nothing about FGS”: A qualitative assessment of knowledge gaps about female genital schistosomiasis among communities living in schistosoma haematobium endemic districts of Zanzibar and Northwestern Tanzania. PLoS Negl Trop Dis [Internet]. 2021;15(9):1–25. Available from: http://dx.doi.org/10.1371/journal.pntd.0009789
